# Simultaneous Characterization and Quantification of Varied Ingredients from *Sojae semen praeparatum* in Fermentation Using UFLC–TripleTOF MS

**DOI:** 10.3390/molecules24101864

**Published:** 2019-05-15

**Authors:** Chuan Chai, Xiaobing Cui, Chenxiao Shan, Sheng Yu, Xinzhi Wang, Hongmei Wen

**Affiliations:** School of Pharmacy, Nanjing University of Chinese Medicine, Nanjing 210029, Jiangsu, China; echo_0523@hotmail.com (C.C.); xiaobingcui@163.com (X.C.); thomastiger@163.com (C.S.); yusheng1219@163.com (S.Y.); wxzatnj@sina.com (X.W.)

**Keywords:** *Sojae semen praeparatum* (SSP), fermentation, conversion, ultra-fast liquid chromatography (UFLC)–TripleTOF MS, principal component analysis (PCA)

## Abstract

Systematic comparison of active ingredients in *Sojae semen praeparatum* (SSP) during fermentation was performed using ultra-fast liquid chromatography (UFLC)–TripleTOF MS and principal component analysis (PCA). By using this strategy, a total of 25 varied compounds from various biosynthetic groups were assigned and relatively quantified in the positive or negative ion mode, including two oligosaccharides, twelve isoflavones, eight fatty acids, *N*–(3–Indolylacetyl)–dl–aspartic acid, methylarginine, and sorbitol. Additionally, as the representative constituents, six targeted isoflavones were sought in a targeted manner and accurately quantified using extracted ion chromatograms (XIC) manager (AB SCIEX, Los Angeles, CA, USA) combined with MultiQuant software (AB SCIEX, Los Angeles, CA, USA). During the fermentation process, the relative contents of oligoses decreased gradually, while the fatty acids increased. Furthermore, the accurate contents of isoflavone glycosides decreased, while aglycones increased and reached a maximum in eight days, which indicated that the ingredients converted obviously and regularly throughout the SSP fermentation. In combination with the morphological changes, which meet the requirements of China Pharmacopoeia, this work suggested that eight days is the optimal time for fermentation of SSP from the aspects of morphology and content.

## 1. Introduction

Fermentation is one of the major processes used in the production of food from soybeans and has played an important role in human life for centuries [1,2]. Many studies have reported the components that are converted and how bioactivities increased in soybean products during the fermentation process [3,4,5,6,7].

*Sojae semen praeparatum* (SSP), whose Chinese herbal name is dandouchi, which is a product of Chinese fermented preparation obtained from the ripe seed of soybean (*Glycine max* (L.) Merr.), has been used as an important component in traditional diets and as an effective traditional Chinese medicine (TCM) among the Chinese community worldwide. More people are expected to consume SSP if the fermentation process includes quality assessment and quality control. Other studies have focused on the active ingredients in SSP, such as isoflavone [8,9,10,11], peptides [12], biogenic amines [13,14], and volatile components [15], the physiological properties of SSP such as anti-oxidative activity [16], anti-proliferative activity [17], anti-α-glucosidase activity [18] and anti-hypertensive effects [19], and species and quantities of fermenting bacteria in SSP spontaneous fermentation, such as bacterial fermentation and fungus fermentation [20,21]. However, no systematic comparison has been conducted of the active ingredients among the raw materials and the SSP products collected at different fermentation stages.

Isoflavones were reported to be representative constituents affecting soybean due to their significant estrogen-like bioactivity [22,23], which increased after fermentation [24,25]. The main isoflavones found in soybean are daidzein, genistein and glycitein, which are present either in glycosidic or aglycone form, mainly with β–glycosides and some 6″–*O*–malonyl or 6″–*O*–acetylglucose [26,27]. Aglyca were reported to have more bioactivity compared to the corresponding glycosides [28]. Some studies suggested that bacterial or fungal β–glycosidases are attractive candidates for use in converting β–glycosides isoflavone to their aglycones, thus enhancing the nutritional value of soy products [29,30]. However, the composition and contents change trends of isoflavones contained in SSP during fermentation have not yet been reported.

Therefore, we aimed to characterize the conversion of ingredients associated with the SSP fermentation process using ultra–fast liquid chromatography-triple time of flight mass spectrometry (UFLC–TripleTOF MS) [31], and accurately quantify the major components that vary using extracted ion chromatograms (XIC) manager (AB SCIEX, Los Angeles, CA, USA) with standard injections, thereby providing some technological supports for the optimization and quality control of SSP fermentation.

## 2. Results and Discussion

### 2.1. Morphologic Changes

**Figure 1:**
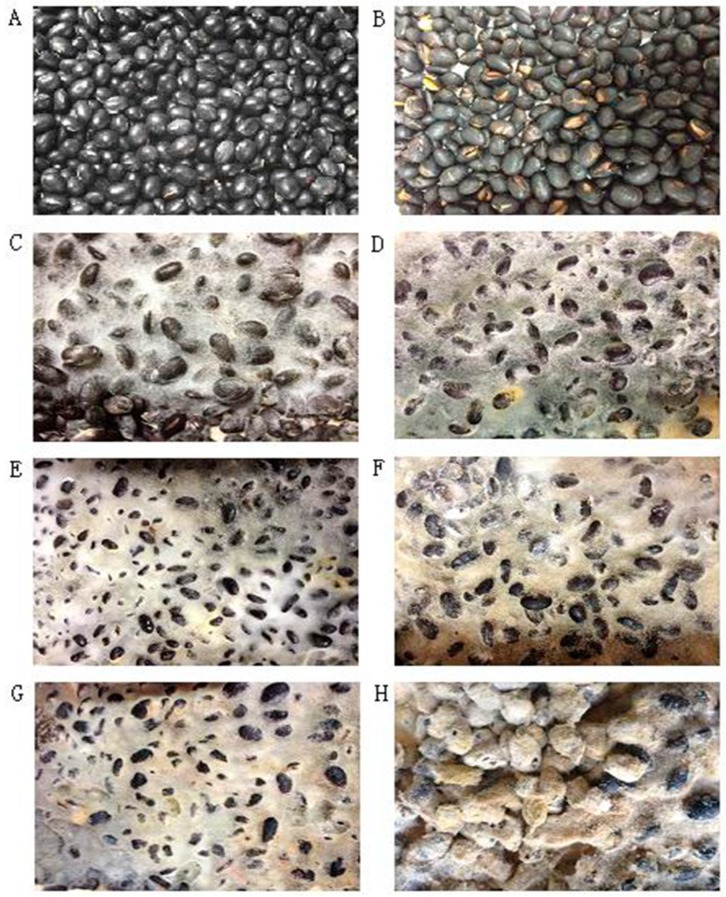
The morphology of soybean (**A**), S0 (**B**), S2 (**C**), S4 (**D**), S6 (**E**), S8 (**F**), S10 (**G**), S15 (**H**) days.

Morphological changes in soybean and SSP products during fermentation were shown in Figure 1. With the increase in fermentation time, black soybean was overgrown with white hyphae, which then changed to yellow in SSP fermented for six days (S6), turned yellow completely in SSP fermented for eight days (S8), and finally hardened. In accordance with the 2015 edition of China Pharmacopoeia [32], moisturized soybeans should be fermented with boiled Artemisiae annuae herba and Mori folium until “yellow cladding”. The morphological changes in S8 were consistent with these requirements, so we speculated that eight days is the optimal time for the fermentation of SSP.

### 2.2. Qualitative Analysis and Principle Component Analysis (PCA)

Using UFLC–TripleTOF MS analysis, information on intact precursors and fragment ions were obtained from a single injection.

**Figure 2:**
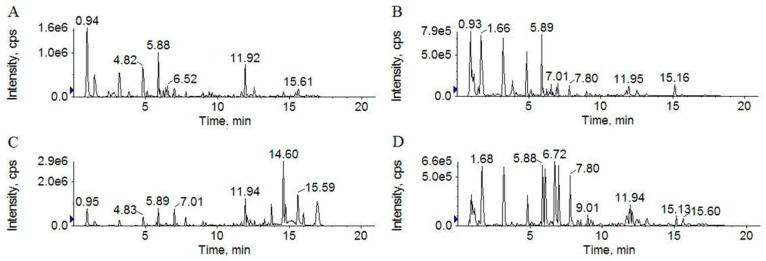
UFLC–TripleTOF MS base peak chromatograms (BPCs) of soybean in positive (**A**) and negative (**B**) ion modes and S8 in positive (**C**) and negative (**D**) ion modes.

The base peak chromatograms (BPCs) of soybean and S8 using both positive and negative ion modes are shown in Figure 2. The BPCs of S8 were significantly different from those of soybeans. Compared to the BPC of soybean in positive ion mode (Figure 2A), the BPC of S8 showed much higher peak intensities at t_R_ = 10–20 min (Figure 2C). Much higher peak intensities occurred at t_R_ = 0–10 min of the BPC for S8 (Figure 2D) in negative ion mode compared to soybean (Figure 2B), which shows that the ingredients converted during the SSP fermentation.

**Figure 3:**
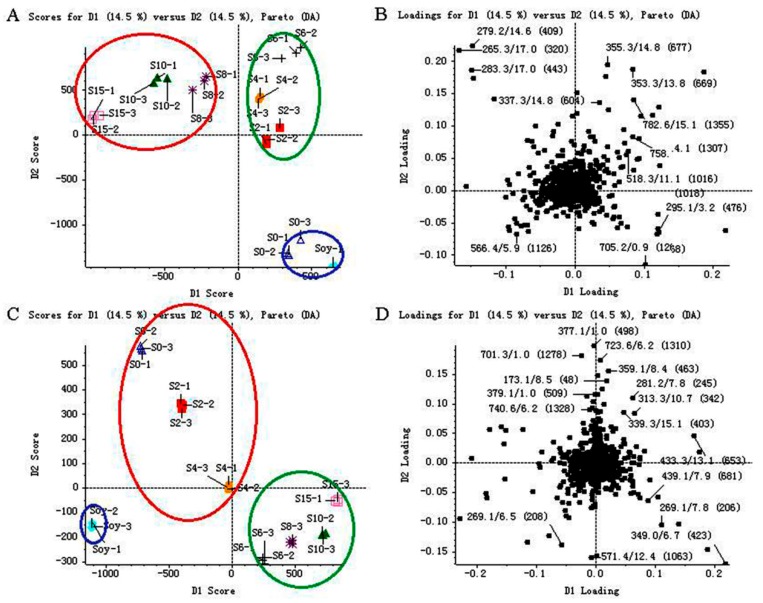
Score plots (**A**,**C**) and loading plots (**B**,**D**) of metabolites determined in soybean (Soy) and *Sojae semen praeparatum* products (S0, S2, S4, S6, S8, S10 and S15) by UFLC–TripleTOF MS in both positive (A and B) and negative (C and D) ion modes. The three clusters (green, blue and red) were used for the color coding of different groups separated by the first and second principal components.

Figure 3A shows that the score plot of soybean and SSP products in positive ion mode is separated into three significant clusters (*P* < 0.05) for the first and the second principal components (PCs). Here, the green cluster (S2 (SSP fermented for two days), S4 (SSP fermented for four days) and S6) and the red cluster (S8, S10 (SSP fermented for ten days) and S15 (SSP fermented for fifteen days)) are separated by the first PC, whereas the blue cluster (soybean and S0) and the green cluster (S2, S4 and S6) are separated by the second PC. The first and second PCs’ values are both 14.5%.

Similarly, Figure 3C illustrates the score plot of soybean and SSP products in negative ion mode, which is separated into three significant clusters for the first and the second PCs. The blue cluster (soybean) and the red cluster (S6, S8, S10 and S15) are separated by the first PC, whereas the blue cluster (soybean) and the green cluster (S0, S2 and S4) are separated by the second PC. The first and second PCs’ values are both 14.5%.

From the corresponding loadings plots (Figure 3B,D), a significant number of variables are located around the observations of the samples, indicating that SSP converted significantly throughout the fermentation process.

The ion species, retention times, molecular formulas, mean measured mass, mass accuracies and assigned identities of the significantly variables are shown in Table 1. [M + H]^+^, [M + Na]^+^, [M + K]^+^, [M + NH_4_]^+^, [M + H + CH_3_OH]^+^, and [M + H − H_2_O]^+^ ion species were found in positive ion mode and [M − H]^–^, [M −H − H_2_O]^−^, [M + CH_3_COO]^–^, and [M + HCOO]^–^ were found in negative ion mode.

We found 29 components of varied classes in positive ion mode, and 22 of them were inferred to be raffinose (**2**), stachyose (**3**), *N*–(3–Indolylacetyl)–dl–aspartic acid (**5**), daidzin (**6**), glycitin (**7**), genistin (**8**), 6″–*O*–malonyldaidzin (**9**), 6″–*O*–malonylglycitin (**10**), 6″–*O*–acetyldaidzin (**11**), 6″–*O*–acetylglycitin (**12**), 6″–*O*–malonylgenistin (**13**), daidzein (**14**), 6″–*O*–acetylgenistin (**15**), glycitein (**16**), methylarginine (**17**), genistein (**18**), dimorphecolic acid (**20**), α–linolenic acid (**22**), linoleic acid (**24**), oleic acid (**25**), palmitic acid (**27**) and stearic acid (**28**) with the help of Peakview^®^ software (AB SCIEX, Los Angeles, CA, USA). We identified 23 of varied classes in negative ion mode, of which 10 were putatively identified as stachyose (**3**), sorbitol (**32**), *N*–(3–Indolylacetyl)–dl–aspartic acid (**5**), daidzin (**6**), genistin (**8**), 6″–*O*–acetyldaidzin (**11**), 6″–*O*–acetylgenistin (**15**), glycitein (**16**), gheddic acid (**42**) and nonadecanoic acid (**43**) by linking the masses of ions to structures. We found 7 assigned and 1 unassigned variable in both positive and negative ion modes.

**Figure 4:**
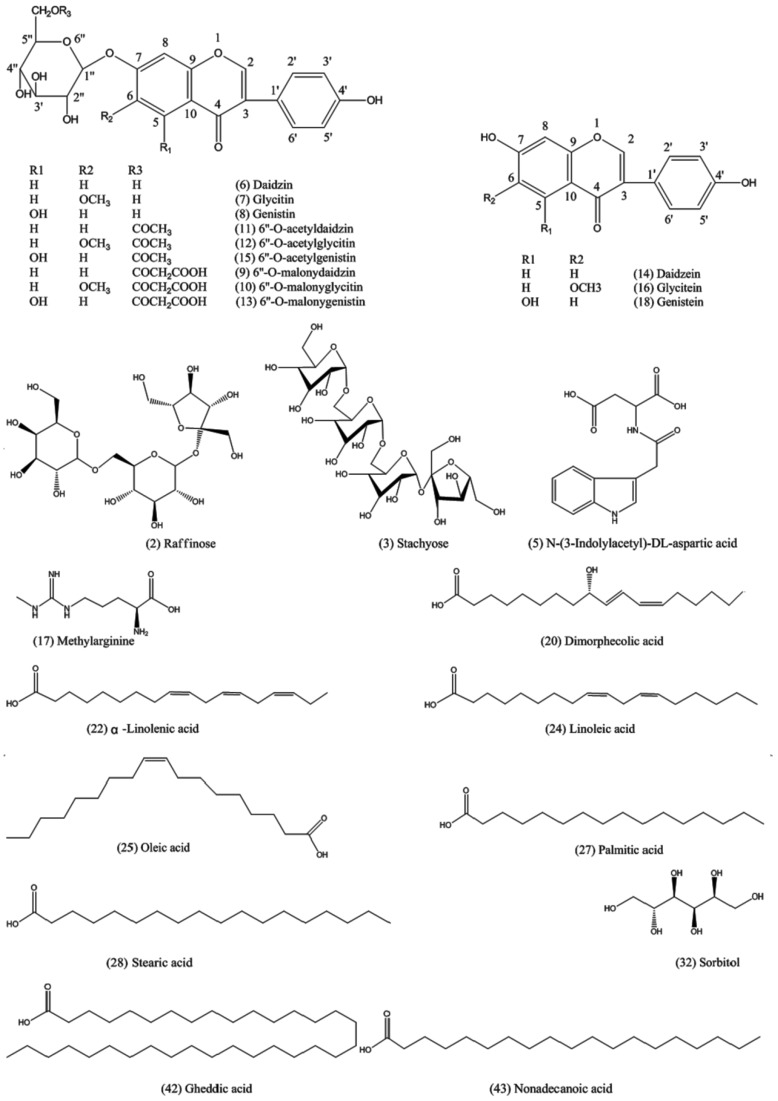
Chemical structures of assigned compounds.

All chemical structures, selected ion intensity trend plots, mass spectra, and mass spectral interpretation of putatively assigned identities were listed in Figure 4, Figures S1 and S2, and Table 2.

Compounds **2** and **3** were inferred as raffinose and stachyose, respectively, for the loss of aglyca. Compounds **14**, **16** and **18** were putatively identified as isoflavone aglycones for their fragment ions at *m*/*z* 137, 153 and 169, respectively, after retro–Diels–Alder reaction. Compounds **6**–**8** were assigned as isoflavone glycosides for their glycones [M + H − glu + H_2_O]^+^ at *m*/*z* 255, 271 and 285 and [M − H − glu + H_2_O]^−^ at *m*/*z* 253, 269 and 283 after deglycosylation. Further dissociation of the glycones yielded a serial of fragments in agreement with the aglycones. The six isoflavone compounds were also confirmed by injecting a mix of standard solutions (Figure 6). Compounds **9**, **10** and **13** were confirmed as isoflavone glycoside malonates: compounds **11**, **12** and **15** were identified as isoflavone acetyl glycosides for their common glycones in comparison with glycosides. Compounds **20**, **22**, **24**, **25**, **27** and **28** were assumed to be a series of fatty acids for the homologous fragment ions at *m*/*z* (67, 81, 95, 109 and 123); (83, 97 and 111); and (57, 71 and 85), the difference between every pair of the fragment ions was 14 (–CH_2_–). Twenty of the assigned compounds were previously reported in soybean [33,34,35,36,37]. However, *N*–(3–Indolylacetyl)–dl–aspartic acid, methylarginine, dimorphecolic acid, gheddic acid, and nonadecanoic acid have never been reported in soybean; sorbitol was only detected in germinating soybean seeds [35], and gheddic acid was identified in *Mori folium* [36]. Six constituents were presumed to be introduced from processing adjuvants or produced during the SSP fermentation process.

### 2.3. Relative Quantitative Analysis

In agreement with previous results [38,39,40], the relative contents of raffinose and stachyose decreased gradually during the entire fermentation process due to degradation by bacteria. As stachyose and raffinose cause indigestion and flatulence in animals after ingestion, the reduction of oligosaccharides is an indication that fermentation can promote the absorption of soybean nutrients. Isoflavone was inferred to be the principle difference among the products obtained from the SSP fermentation process due to its high proportions of varied components. With increasing fermentation time, the relative contents of isoflavone glycosides decreased while the isoflavone aglycones increased, reaching a maximum in S8. The isoflavone glycoside malonates decreased while the isoflavone acetyl glycosides increased to a maximum in S2 and then dropped. We assumed that the isoflavone glycoside malonates were transformed to acetyl glycosides in the early fermentation period due to their heat instability, and that all the isoflavone glycosides were converted to isoflavone aglycones. The increase in fatty acids showed that lipids could be degraded during fermentation. All the results provide some technological supports for the optimization and quality control of SSP fermentation. Given of the regular component conversion in SSP during fermentation, further identification of the unassigned varied ingredients present in SSP is needed.

### 2.4. Accurate Quantitative Analysis

XIC manager combined with MultiQuant software was used to automatically highlight all findings above a defined thresholds at an exactive mass of ± 0.02 Da and to quantitatively compare samples with a series of standard injections.

The validation values are summarized in Table 3. The calibration curves show satisfactory linearity. The correlation coefficient (*r*) ranged from 0.9840–0.9981 for all the isoflavones. Limit of detection (LOD) and limit of quantitation (LOQ) values were 0.1–50.0 and 2.0–250.0 ng/mL, respectively. The intra and inter-day precisions were less than 0.48% and 2.87%, respectively. The repeatability was within 2.53–4.82%. The recoveries were between 97.61 ± 3.73% and 104.84 ± 2.58% at different spiking concentration levels. The short-term stability analyzed at various periods was less than 4.10%. The above results demonstrate that the established method is accurate and reproducible for determining the six isoflavones in SSP.

**Figure 5:**
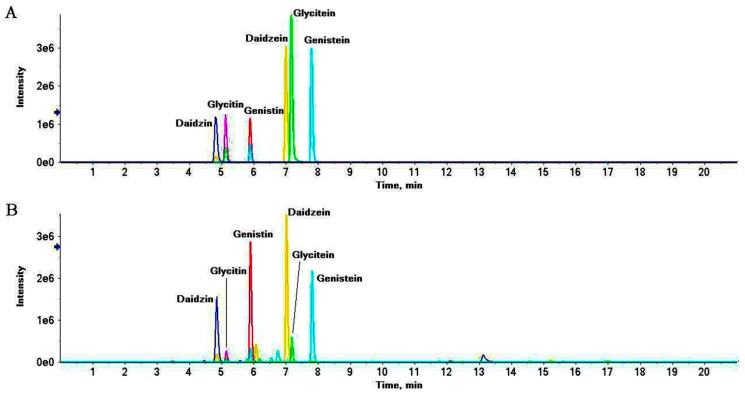
Representative extract ions chromatograms (XIC) of mix standard solution (**A**) and S8 (**B**) at *m*/*z* 417.118 ± 0.02 (daidzin), 447.129 ± 0.02 (glycitin), 433.113 ± 0.02 (genistin), 255.065 ± 0.02 (daidzein), 285.076 ± 0.02 (glycitein) and 271.060 ± 0.02 (genistein).

**Figure 6:**
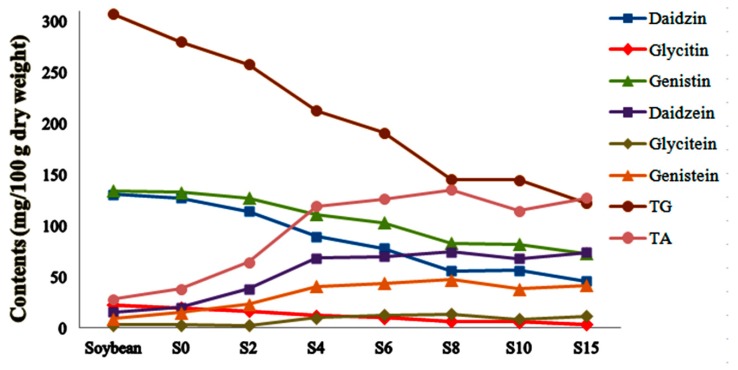
Content profile plots for six isoflavones, total glycoside (TG), and aglycone (TA) in SSP products collected at different fermentation stages.

Controlled by mix standard solution, the accurate quantitative results of six isoflavones in SSP were summarized in Figure 5. All six isoflavones were identified in soybean and SSP products. With increasing fermentation time, daidzin, glycitin and genistin decreased while daidzein, glycitein and genistein increased and raised to the top in S8 at 74.50, 13.52 and 47.42 mg/100 g dry weight respectively. Total glycoside and aglycone were also calculated as the sum of each individual isoflavone and presented in Figure 6. As the fermentation time increased, total glycoside contents decreased, while total aglycone contents increased significantly and rose to the top in the S8. The total glycoside content in S8 was less than half a percent of soybean’s while the total aglycone content in S8 was 4.8 times higher than that in soybean, indicating that the ingredients converted regularly during the fermentation process.

Previous research has suggested that the increase in aglycone content and β−glucosidase activity during the fermentation of soybean show a similar trend [41]. As an attractive candidate to convert isoflavone glycosides to their aglycones, β−glucosidase reached its maximum activity on the eighth days of fermentation.

As aglycone possesses higher bioactivity and bioavailability compared to the β−glycosides isoflavone, the quantitative results of the representative constituents illustrated that eight days is the optimal time for the fermentation of SSP, which agrees with the morphologic changes (Figure 1). We reasoned that the bioactivities may be related to the variations in isoflavone content and β−glucosidase activity during SSP fermentation. Moreover, we created a perfect setup for SSP fermentation quality assessment and quality control. To determine the impact of bacterial or fungi on SSP fermentation, −further study is required.

## 3. Materials and Methods

### 3.1. Chemicals and Reagents

The reference standards of daidzin, glycitin, genistin, daidzein, glycitein and genistein were all purchased from Sigma−Aldrich (St. Louis, MO, USA)

Liquid chromatography (LC)/MS−grade acetonitrile, formic acid, methanol, and water were purchased from Merck Co. (Darmstadt, Germany).

Soybean, Artemisiae annuae herba and Mori folium used in the fermentation were purchased from YiFeng TCM shop (Nanjing, China) and authenticated by Associate Professor Jianwei Chen (Department of Pharmacy, Nanjing University of Chinese Medicine, Nanjing, Jiangsu, China).

### 3.2. SSP Fermentation

**Figure 7:**
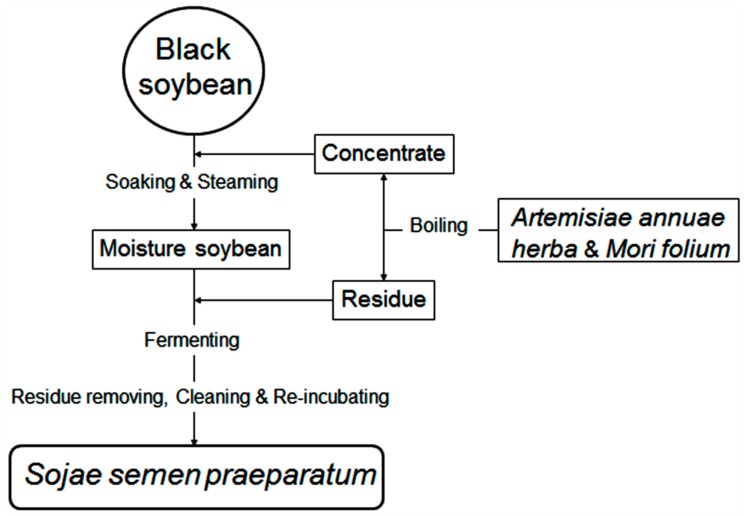
Flow diagram for fermentation of soybean to *Sojae semen praeparatum* (SSP).

SSP was fermented in the laboratory and the preparation was performed as described in detail by the 2015 edition of China Pharmacopoeia as illustrated in Figure 7. The steps and parameters were as follows:

Artemisiae annuae herba (100 g dry weight) and Mori folium (90 g dry weight) were washed and boiled with water (3600 mL) for 1 h in triplicate, and the decoction was concentrated to a relative density of 1.10–1.12 g/cm^3^ concentrate. Black soybean (1000 g dry weight) was soaked in the concentrate overnight and steamed for 1.5 h while covered with wet cheesecloth. Aliquots (100 g wet weight) of the moisture soybean were placed on enamel trays covered with wet cheesecloth, which were covered with the residue of boiled Artemisiae annuae herba and Mori folium and incubated at 37 °C with 60–80% humidity. After fermenting for 0, 2, 4, 6, 8, 10, 15 days and removal of residues, wash cleaning, and re-incubating until a sweet smell drifting out, the SSP samples (S0, S2, S4, S6, S8, S10, S12, and S15) were then dried and pulverized into powder using an electric mill and sieved through 80 mesh sieves.

### 3.3. Sample Extraction

One gram of the powdered samples was accurately weighed and extracted with 25 mL of 75% methanol at 80 °C for 30 min using a Soxhlet extractor. This was followed by centrifugation for 15 min at 12000 rpm. The extraction supernatants were then diluted 10 times and filtered through a 0.45 µm filter unit.

### 3.4. Standard Solutions Preparation

Mix standard solutions were prepared by accurately weighing the standard substances and mixing them in 75% methanol. This standard mixture was filtered through a 0.45 µm filter unit.

### 3.5. LC-MS Spectrometric Conditions

An ultra-fast liquid chromatography system (Shimadzu Corporation UFLC XR; Kyoto, Japan) was connected to a triple time−of−flight mass spectrometer (TripleTOF 5600 system, AB SCIEX, Los Angeles, CA, USA) with an electrospray ionization source.

All samples were separated by an ACQUITY UPLC BEH C_18_ column (2.1 mm × 100 mm, 1.7 µm, Waters Corp., Milford, MA, USA). A binary solvent gradient consisting of solvent A (water with 0.2% formic acid) and solvent B (acetonitrile with 0.2% formic acid) was used. The flow rate was 300 µL/min. The total run time was 21 min with a gradient as follows: 0–3 min, 10–16% B; 3–7 min, 16–50% B; 7–12 min, 50–80% B; 12–15 min, 80–90% B; 15–17 min, 90–10% B; and 17–21 min, 10% B for column equilibration before the next run. An injection volume of 1 µL was used. The column temperature was 40 °C.

The samples were analyzed by acquiring full scan MS data in both positive and negative ion modes. The automatic data-dependent information product ion spectra (IDA−MS/MS) without any predefinition of the ions was checked. A calibrated delivery system was used to ensure the accuracy error of masses less than 1 ppm. The settings were nitrogen gas for nebulization at 55 psi, heater gas pressure at 55 psi, curtain gas at 35 psi, temperature of 500 °C, and ion spray voltage at 5500 V in positive ion mode, and −4500 V in negative ion mode. The acquisition of a survey tripleTOF MS spectrum was operated under high-resolution settings. The optimized declustering potential (DP) and collision energy (CE) were set at 80 eV and 15 eV in positive ion mode, and to −80 eV and −15 eV in negative ion mode, respectively. A sweeping collision energy setting at 35/−35 eV ± 15 eV was applied for collision-induced dissociation (CID).

### 3.6. Method Validation

The method was fully validated in accordance with guidelines on linearity, precision, recovery, detection limit, quantification limit, and stability. Calibration curves were generated by plotting peak area against the concentration of standard solutions. The intra-day precision was examined for six replicates of injections with the mixed standard solutions in one day, and the inter-precision was determined by injection in duplicates over three consecutive days. All the results are expressed using the relative standard deviation (RSD). The LOD and LOQ were calculated based on the peak−to−noise ratios of 3:1 and 10:1, respectively. The repeat, recovery, and stability were tested on the analytes in S8, and the repeatability was analyzed on six sample solutions from the same sample in parallel. The recovery was used to evaluate the accuracy at different spiking concentration levels (80%, 100%, and 120% as compared to the nominal concentration) of standard solutions. and the sample stability was tested by periodic analysis at room temperature for various periods (0, 2, 4, 8, 12, 16, 20, and 24 h).

### 3.7. Data Processing

TOF−MS data were collected using Analyst^®^ version 1.6 software (AB SCIEX, Los Angeles, CA, USA) and processed using PeakView^®^ version 1.2 software (AB SCIEX, Los Angeles, CA, USA) with the XIC Manager (AB SCIEX, Los Angeles, CA, USA) add-in and MultiQuant™ version 2.1 software (AB SCIEX, Los Angeles, CA, USA).

The PeakView^®^ software contained a simple fragment ion predictor to help link the MS/MS spectrum to structures (saved as .mol files) and to provide insights into fragmentation mechanisms.

XIC Manager was used for targeted and non-targeted data processing, which consisted of a table for defining a list of masses or formulae to generate extracted ion chromatograms (XIC), and to review the identification of detected compounds. Our high confidence in results is based on retention times, accurate mass, isotopic pattern and MS/MS library searching.

The PCA was performed using MarkerView^®^ software, where three repeated spectra for each sample were imported and analyzed with Pareto scaling. The *T*−value and corresponding *P*−value were calculated between each group and all the other eight groups. The program was linked back to the raw data so that differences could be directly visualized in spectra or chromatograms. The converted components were putatively identified by PeakView^®^ software.

## 4. Conclusions

This was the first systematic comparison of active ingredients in the raw materials and processed products obtained during *Sojae semen praeparatum* (SSP) fermentation. Simultaneous characterization and quantification were performed using ultra-fast liquid chromatography (UFLC)−TripleTOF MS combined with XIC manager. The quantitative results verified that the components converted during the SSP fermentation, and we identified 45 components in positive ion mode and negative ion mode, in which 25 were putatively identified and a high proportion was isoflavone. *N*−(3−Indolylacetyl)−dl−aspartic acid, methylarginine, dimorphecolic acid, sorbitol, gheddic acid, and nonadecanoic acid were presumed to be introduced from processing adjuvants or produced during the fermentation process. The relative contents of raffinose and stachyose decreased gradually, while the fatty acids and isoflavone aglycones increased, which indicated that fermentation promotes the absorption of soybean nutrition and lipids degradation. The accurate quantitation of isoflavone, the representative constituents in soybean, revealed that fermentation for eight days produced a marked increase in the content of aglycone, the bioactive isoflavone, and a significant reduction in the content of β−glycosides isoflavone compared with unfermented soybean. This illustrated that eight days is the optimal time for the fermentation of SSP from the aspects of content, in agreement with the morphologic changes. We reasoned that the bioactivities of SSP might be related to isoflavone. Our study has provided some technological support for the optimization and quality control of SSP fermentation.

## Figures and Tables

**Table 1:** Varied components putatively identified from soybean and *Sojae semen praeparatum* products in both positive and negative ion modes.

Ionization Mode	Compound No.	t_R_^a^ (min)	Molecular Formula	Mass (Da)	Ion Species	Mean Measured Mass (Da)	Mass Accuracy (ppm)	Assigned Identity	References
Positive	**1**	0.93	C_12_H_18_N_6_O_6_	342.1288	[M + K]^+^	381.0907	−0.4	/ ^b^	– ^c^
	**2**	0.93	C_18_H_32_O_16_	504.1690	[M + K]^+^	543.1474	−1.9	Raffinose	[33]
	**3**	0.95	C_24_H_42_O_21_	666.2213	[M + K]^+^	705.2045	−1.2	Stachyose	[33]
	**4**	1.44	C_13_H_15_N_3_O_5_	293.1012	[M + NH_4_]^+^	311.1328	−2.2	/ ^b^	− ^c^
	**5**	3.86	C_14_H_14_N_2_O_5_	290.0903	[M + H]^+^	291.0955	−4.6	*N*−(3−Indolylacetyl)−dl−aspartic acid	− ^c^
	**6**	4.81	C_21_H_20_O_9_	416.1107	[M + H]^+^	417.1307	0.8	Daidzin	[37]
	**7**	5.14	C_22_H_22_O_10_	446.1213	[M + H]^+^	447.1429	−0.3	Glycitin	[37]
	**8**	5.89	C_21_H_20_O_10_	432.1057	[M + H]^+^	433.1266	0.9	Genistin	[37]
	**9**	6.03	C_24_H_22_O_12_	502.1111	[M + H]^+^	503.1335	−0.6	6″−*O*−malonyldaidzin	[37]
	**10**	6.04	C_25_H_24_O_13_	532.1217	[M + H]^+^	533.1451	−1.3	6″−*O*−malonylglycitin	[37]
	**11**	6.44	C_23_H_22_O_10_	458.1213	[M + H]^+^	459.1435	−0.2	6″−*O*−acetyldaidzin	[37]
	**12**	6.51	C_24_H_24_O_11_	488.1319	[M + H]^+^	489.1535	−1.0	6″−*O*−acetylglycitin	[37]
	**13**	6.57	C_24_H_22_O_13_	518.1060	[M + H]^+^	519.1285	−0.8	6″−*O*−malonylgenistin	[37]
	**14**	7.00	C_15_H_10_O_4_	254.0579	[M + H]^+^	255.0726	0.9	Daidzein	[37]
	**15**	7.05	C_23_H_22_O_11_	474.1162	[M + H]^+^	475.1379	−1.0	6″−*O*−acetylgenistin	[37]
	**16**	7.17	C_16_H_12_O_5_	284.0685	[M + H]^+^	285.0840	0.3	Glycitein	[37]
	**17**	7.69	C_7_H_16_N_4_O_2_	188.1268	[M + H + H_2_O]^+^	207.1452	−3.9	Methylarginine	− ^c^
	**18**	7.80	C_15_H_10_O_5_	270.0528	[M + H]^+^	271.0680	1.0	Genistein	[37]
	**19**	11.94	C_21_H_45_N_9_O_6_	519.3493	[M + H]^+^	520.3534	1.0	/ ^b^	− ^c^
	**20**	13.29	C_18_H_32_O_3_	296.2710	[M + H]^+^	297.2517	−3.9	Dimorphecolic acid	− ^c^
	**21**	13.77	C_23_H_44_O_2_	352.3336	[M + H]^+^	353.2797	0.1	/ ^b^	− ^c^
	**22**	14.61	C_18_H_30_O_2_	278.2246	[M + H]^+^	279.2408	−0.4	α−Linolenic acid	[34]
	**23**	14.76	C_23_H_46_O_2_	354.3492	[M + H] ^+^	355.2950	0.8	/ ^b^	− ^c^
	**24**	15.62	C_18_H_32_O_2_	280.2402	[M + H]^+^	281.2567	0.7	Linoleic acid	[34]
	**25**	15.70	C_18_H_34_O_2_	282.2559	[M + H + CH_3_OH]^+^	315.2915	0.6	Oleic acid	[34]
	**26**	16.01	C_21_H_40_O_2_	324.3023	[M + H + CH_3_OH]^+^	357.3101	2.9	/ ^b^	− ^c^
	**27**	16.67	C_16_H_32_O_2_	256.2402	[M + H]^+^	257.2565	−0.3	Palmitic acid	[34]
	**28**	17.04	C_18_H_36_O_2_	284.2715	[M + H]^+^	285.3050	−0.8	Stearic acid	[34]
Negative	**29**	0.92	C_12_H_18_N_6_O_6_	342.1288	[M + HCOO]^−^	387.1623	1.7	/ ^b^	− ^c^
	**3**	0.95	C_24_H_42_O_21_	666.2213	[M + HCOO]^−^	711.3077	1.3	Stachyose	[33]
	**30**	1.05	C_30_H_32_N_6_O_9_	620.2220	[M – H − H_2_O]^−^	601.2133	−1.1	/ ^b^	− ^c^
	**31**	3.12	C_16_H_18_N_6_	294.1582	[M − H]^−^	293.1552	−1.4	/ ^b^	− ^c^
	**32**	3.21	C_6_H_14_O_6_	182.0790	[M + CH_3_COO]^−^	241.0915	0.8	Sorbitol	[35]
	**5**	3.85	C_14_H_14_N_2_O_5_	290.0903	[M − H]^−^	289.1078	−1.3	*N*−(3−Indolylacetyl)−dl−aspartic acid	− ^c^
	**6**	4.81	C_21_H_20_O_9_	416.1107	[M + HCOO]^−^	461.1650	0.2	Daidzin	[37]
	**33**	5.64	C_18_H_10_N_2_O_6_	350.0528	[M − H]^−^	349.0451	−2	/ ^b^	− ^c^
	**8**	5.89	C_21_H_20_O_10_	432.1057	[M + HCOO]^−^	477.1629	−2.5	Genistin	[37]
	**34**	6.15	C_18_H_4_N_6_	304.0497	[M + CH_3_COO]^−^	363.0635	−0.5	/ ^b^	− ^c^
	**11**	6.42	C_23_H_22_O_10_	458.1213	[M + HCOO]^−^	503.1805	−2.3	6″−*O*−acetyldaidzin	[37]
	**35**	6.47	C_13_H_10_O_6_	262.0466	[M − H]^−^	261.0400	2.4	/ ^b^	− ^c^
	**36**	6.68	C_19_H_6_N_6_O_3_	366.0490	[M − H]^−^	365.0430	−1.3	/ ^b^	− ^c^
	**15**	7.04	C_23_H_22_O_11_	474.1162	[M + HCOO]^−^	519.1784	2.3	6″−*O*−acetylgenistin	[37]
	**16**	7.15	C_16_H_12_O_5_	284.0685	[M − H]^−^	283.0969	2.1	Glycitein	[37]
	**37**	7.91	C_18_H_18_O_10_	394.0895	[M + HCOO]^−^	439.0878	−2.0	/ ^b^	− ^c^
	**38**	10.83	C_31_H_50_N_2_O_9_	594.3505	[M − H]^−^	593.3490	−0.6	/ ^b^	− ^c^
	**39**	11.29	C_19_H_36_O_3_	312.2654	[M – H − H_2_O]^−^	293.2483	1.7	/ ^b^	− ^c^
	**40**	11.66	C_32_H_48_N_6_O_5_	596.3675	[M − H]^−^	595.3613	1.9	/ ^b^	− ^c^
	**41**	12.06	C_17_H_36_N_8_O_5_	432.2798	[M − H]^−^	431.2728	0.1	/ ^b^	− ^c^
	**42**	12.54	C_34_H_68_O_2_	508.3389	[M − H]^−^	507.3340	0.1	Gheddic acid	[36]
	**43**	12.58	C_19_H_38_O_2_	298.2861	[M − H]^−^	297.2814	4.0	Nonadecanoic acid	− ^c^
	**44**	13.57	C_18_H_42_N_10_O	414.3543	[M + HCOO]^−^	459.3520	−1.3	/ ^b^	− ^c^
	**45**	15.55	C_12_H_28_N_8_O_6_	380.2121	[M − H]^−^	379.2049	−2.6	/ ^b^	− ^c^

^a^ t_R_, retention time; ^b^ Not assigned; ^c^ No reference.

**Table 2:** Mass spectral interpretation of assigned compounds in soybean and *Sojae semen praeparatum* products.

Compound No.	Assigned Identity (Ion Mode)	MS/MS Fragments Ions
**2**	Raffinose (+)	543.1474[M + K]^+^, 381.0905[M + K − glu + H_2_O]^+^,
**3**	Stachyose (+)	705.2045[M + K]^+^, 543.1471[M+K − glu]^+^,
	(−)	711.3077[M + HCOO]^−^, 665.3014[M − H]^−^, 485.2130[M – H − glu]^−^, 341.1514[M − H − 2glu + 2H_2_O]^−^,
**5**	*N*−(3−Indolylacetyl)−dl−aspartic acid (+)	291.0955[M + H]^+^, 161.0645[C_5_H_6_O_2_N + H]^+^, 139.0428[M + H − C_8_H_6_N − 2H_2_O]^+^,
	(−)	289.1078[M − H]^−^, 271.0968[M − H − H_2_O]^−^, 245.1127[M − H − CO_2_]^−^, 227.1008[M − H − CO_2_ − H_2_O]^−^,
**6**	Daidzin (+)	439.1128[M + Na]^+^, 417.1307[M + H]^+^, 277.0551[M + Na − glu + H_2_O]^+^, 255.0728[M + H − glu + H_2_O]^+^,
	(−)	461.1650[M + HCOO]^−^, 415.1556[M − H]^−^, 253.0825[M − H − glu + H_2_O]^−^,
**7**	Glycitin (+)	469.1235[M + Na]^+^, 447.1429[M + H]^+^, 307.0662[M + Na − glu + H_2_O]^+^, 285.0839[M + H – glu + H_2_O]^+^,
**8**	Genistin (+)	433.1266[M + H]^+^, 271.0674[M + H – glu + H_2_O]^+^, 243.0712[M + H – glu + H_2_O − CO]^+^, 215.0752[M + H – glu + H_2_O − 2CO]^+^, 153.0218[M + H − C_13_H_12_O_7_]^+^,
	(−)	477.1629[M + HCOO]^−^, 431.1529[M − H]^−^, 269.0795[M – H – glu + H_2_O]^−^,
**9**	6″−*O*−malonyldaidzin (+)	525.1146[M + Na]^+^, 503.1335[M + H]^+^, 481.1244[M + Na − CO_2_]^+^, 439.1133[M + Na − malonyl − H_2_O]^+^, 277.0549[M + Na – malonyl − glu]^+^, 255.0728[M + H – malonyl − glu]^+^,
**10**	6″−*O*−malonylglycitin (+)	533.1451[M + H]^+^, 285.0845[M + H – malonyl − glu]^+^,
**11**	6″−*O*−acetyldaidzin (+)	459.1435[M + H]^+^, 255.0726[M + H – acetyl − glu]^+^,
	(−)	503.1805[M + HCOO]^−^, 457.1720[M − H]^−^, 253.0822[M − H − acetyl − glu]^−^,
**12**	6″−*O*−acetylglycitin (+)	489.1535[M + H]^+^, 285.0839[M + H – acetyl − glu]^+^,
**13**	6″−*O*−malonylgenistin (+)	541.1093[M + Na]^+^, 523.0995[M + Na − H_2_O]^+^, 519.1285[M + H]^+^, 497.1167[M + Na − CO_2_]^+^, 455.1096[M + Na – malonyl − H_2_O]^+^, 293.0505[M + Na – malonyl − glu]^+^, 271.0678[M + H – malonyl − glu]^+^,
**14**	Daidzein (+)	277.0550[M + Na]^+^, 255.0726[M + H]^+^, 237.0611[M + H − H_2_O]^+^, 227.0762[M + H − CO]^+^, 199.0806[M + H − 2CO]^+^, 181.0695[M + H − 2CO − H_2_O]^+^, 137.0273[M + H − C_8_H_6_O]^+^, 91.0582[M + H − H_2_O − C_9_H_6_O_2_]^+^,
**15**	6″−*O*−acetylgenistin (+)	475.1379[M + H] ^+^, 271.0682[M + H − acetyl − glu]^+^,
	(−)	519.1784[M + HCOO]^−^, 473.1689[M − H]^−^, 269.0795[M − H – acetyl − glu]^−^,
**16**	Glycitein (+)	307.0672[M + Na]^+^, 285.0840[M + H]^+^, 270.0599[M + H − CH_3_]^+^, 242.0642[M + H − CH_3_ − CO]^+^, 169.0614[M + H − C_8_H_4_O]^+^, 141.0740[M + H − C_9_H_4_O_2_]^+^,
	(−)	283.0969[M − H]^−^, 268.0714[M − H − CH_3_]^−^, 240.0729[M − H − CH_3_ − CO]^−^, 196.0776[M − C_2_H_3_O − OH − OH]^−^,
**17**	Methylarginine (+)	207.1452[M + H + H_2_O]^+^, 189.1321[M + H]^+^, 161.1377[M + H − H_2_O]^+^,
**18**	Genistein (+)	271.0680[M + H]^+^, 253.0565[M + H − H_2_O]^+^, 243.0716[M + H − CO]^+^, 215.0757[M + H − 2CO]^+^, 153.0223[M + H − C_8_H_6_O]^+^,
**20**	Dimorphecolic acid (+)	297.2517[M + H]^+^, 279.2398[M + H − H_2_O]^+^, 261.2287[M + H − 2H_2_O]^+^, 233.2328[M + H − 2H_2_O − CO]^+^, 109.1051[M + H − C_9_H_17_COOH − CH_4_]^+^, 97.1051[M + H − C_9_H_17_COOH − C_2_H_4_]^+^, 81.0743[M + H − C_9_H_17_COOH − C_3_H_8_]^+^, 67.0590[M + H − C_9_H_17_COOH − C_4_H_10_]^+^
**22**	α−Linolenic acid (+)	297.2517[M + H + H_2_O]^+^, 279.2408[M + H]^+^, 149.0276[M + H − C_6_H_3_COOH]^+^, 135.1206[M + H − C_7_H_15_COOH]^+^, 125.1000[M + H − C_8_H_13_COOH]^+^, 123.1200[M + H − C_8_H_15_COOH]^+^, 109.1045[M + H − C_9_H_17_COOH]^+^, 95.0892[M + H − C_10_H_19_COOH]^+^, 81.0738[M + H − C_11_H_21_COOH]^+^, 67.0588[M + H − C_12_H_23_COOH]^+^,
**24**	Linoleic acid (+)	313.2827[M + H + CH_3_OH]^+^, 281.2567[M + H]^+^, 263.2443[M + H − H_2_O]^+^, 239.2433[M + H + CH_3_OH − H_2_O − C_4_H_8_]^+^, 221.2318[M + H − CH_3_COOH]^+^, 147.1210[M + H + CH_3_OH − C_12_H_22_]^+^ 133.1051[M + H + CH_3_OH − C_13_H_24_]^+^, 109.1049[M + H − C_9_H_19_COOH]^+^, 95.0895[M + H − C_10_H_21_COOH]^+^, 71.0902[M + H − C_12_H_21_COOH]^+^, 57.0755[M + H − C_13_H_23_COOH]^+^,
**25**	Oleic acid (+)	315.2915[M + H + CH_3_OH]^+^, 283.2461[M + H]^+^, 271.5449[M + H + CH_3_OH − CO_2_]^+^, 267.0220[M + H − CH_4_]^+^, 265.2757[M + H − H_2_O]^+^, 187.1151[M + H + CH_3_OH − C_9_H_20_]^+^, 171.0300[M + H − C_8_H_16_]^+^, 114.9656[M + H − C_9_H_15_COOH]^+^, 96.9540[M + H − C_10_H_21_COOH]^+^, 83.0875[M + H − C_11_H_23_COOH]^+^, 57.0750[M + H − C_13_H_25_COOH]^+^
**27**	Palmitic acid (+)	257.2565[M + H]^+^, 201.1894[M + H − C_4_H_8_]^+^, 97.1025[M + H − H_2_O − C_10_H_22_]^+^, 71.0902[M + H − C_10_H_21_COOH]^+^, 57.0753[M + H − C_11_H_23_COOH]^+^,
**28**	Stearic acid (+)	285.3050[M + H]^+^, 267.2656[M + H − H_2_O]^+^, 126.9036[M + H − C_8_H_17_COOH]^+^, 83.0895[M + H − C_10_H_21_COOH − CH_4_]^+^, 69.0739[M + H − C_11_H_23_COOH − CH_4_]^+^, 57.0742[M + H − C_13_H_27_COOH]^+^,
**32**	Sorbitol (−)	241.0915[M + CH_3_COO]^−^, 223.0801[M + CH_3_COO − H_2_O]^−^, 181.0622[M − H]^−^, 149.0803[M – H − CH_3_OH]^−^,
**42**	Gheddic acid (−)	507.3340[M − H]^−^, 279.2671[M – H − C_13_H_27_COOH]^−^, 153.0148[M – H − C_22_H_45_COOH]^−^,
**43**	Nonadecanoic acid (−)	297.2814[M − H]^−^, 279.2672[M – H − H_2_O]^−^, 183.1614[M − H − C_5_H_9_COOH]^−^,

**Table 3:** Validation data of targeted analytes.

Isoflavone	RT ^a^ (min)	Regression Equation ^b^	Linear Range	*r*	LOD ^c^	LOQ ^d^	Precision (RSD % ^e^)		Repeatability (RSD, %)	Recovery (Mean ^f^ ± RSD %)	Stability
			(µg/mL)		(ng/mL)	(ng/mL)	Intra Day	Inter Day			(RSD % ^a^)
Daidzin	4.81	*Y* = 58544*X* + 384206	0.010−100.0	0.9979	5.0	10.0	1.14	4.24	3.98	103.83 ± 3.15	4.10
Glycitin	5.14	*Y* = 49463*X* + 421236	0.010−100.0	0.9981	5.0	10.0	0.57	2.87	3.27	104.84 ± 2.58	1.84
Genistin	5.89	*Y* = 60953*X* +286019	0.100−600.0	0.9980	10.0	50.0	0.48	4.48	4.41	97.76 ± 4.70	3.04
Daidzein	7.02	*Y* = 76972 *X* + 705619	0.100−100.0	0.9840	0.1	3.0	1.61	3.50	2.53	97.61 ± 3.73	3.34
Glycitein	7.17	*Y* = 52697 *X* + 643182	0.010−200.0	0.9973	0.1	2.0	1.42	2.61	4.19	103.71 ± 2.69	3.78
Genistein	7.80	*Y* = 78899 *X* + 342189	0.50−200.0	0.9973	50.0	250.0	2.35	3.99	4.82	99.67 ± 3.16	2.39

^a^ RT, retention time; ^b^ *Y*, peak area; *X*, concentration (µg/mL); ^c^ LOD, Limit of detection (S/N = 3); ^d^ LOQ, Limit of quantification (S/N = 10). ^e^ Relative standard deviation (%) = (standard deviation / mean) × 100. (*n* = 3); ^f^ Mean extraction yield (%) = (detected amount − original amount)/spiked amount × 100. (*n* = 3).

## Supplementary Materials

The following are available online, Figure S1: Selected ion intensity trend plots of assigned identities., Figure S2: Mass spectrum of assigned compounds in soybean and *Semen sojae praeparatum* products.
