# Supplementary material for: Simultaneous Characterization and Quantification of Varied Ingredients from Sojae semen praeparatum in Fermentation Using UFLC–TripleTOF MS
Source: Molecules. 2019 May 15;24(10):1864. doi: 10.3390/molecules24101864 (PMC6571576; doi:10.3390/molecules24101864)
Supplement: Supplementary file 1 [file molecules-24-01864-s001.pdf]

*Supplementary Material*

# Simultaneous characterization and quantification of varied ingredients from *Semen sojae praeparatum* using fermentation by UFLC- TripleTOF MS

Chuan Chai, Xiaobing Cui, Chenxiao Shan, Sheng Yu, Xinzhi Wang, Chunyan Ou, Hongmei Wen*

*School of Pharmacy, Nanjing University of Chinese Medicine, Nanjing 210029, Jiangsu, China*

*Correspondence information: Prof. Hongmei Wen; School of Pharmacy, Nanjing University of Chinese Medicines; Xianlin Avenue No. 138, 210029 Nanjing, Jiangsu ,China.

E-mail: njwenhm@126.com; TEL: +86-25-85811839.

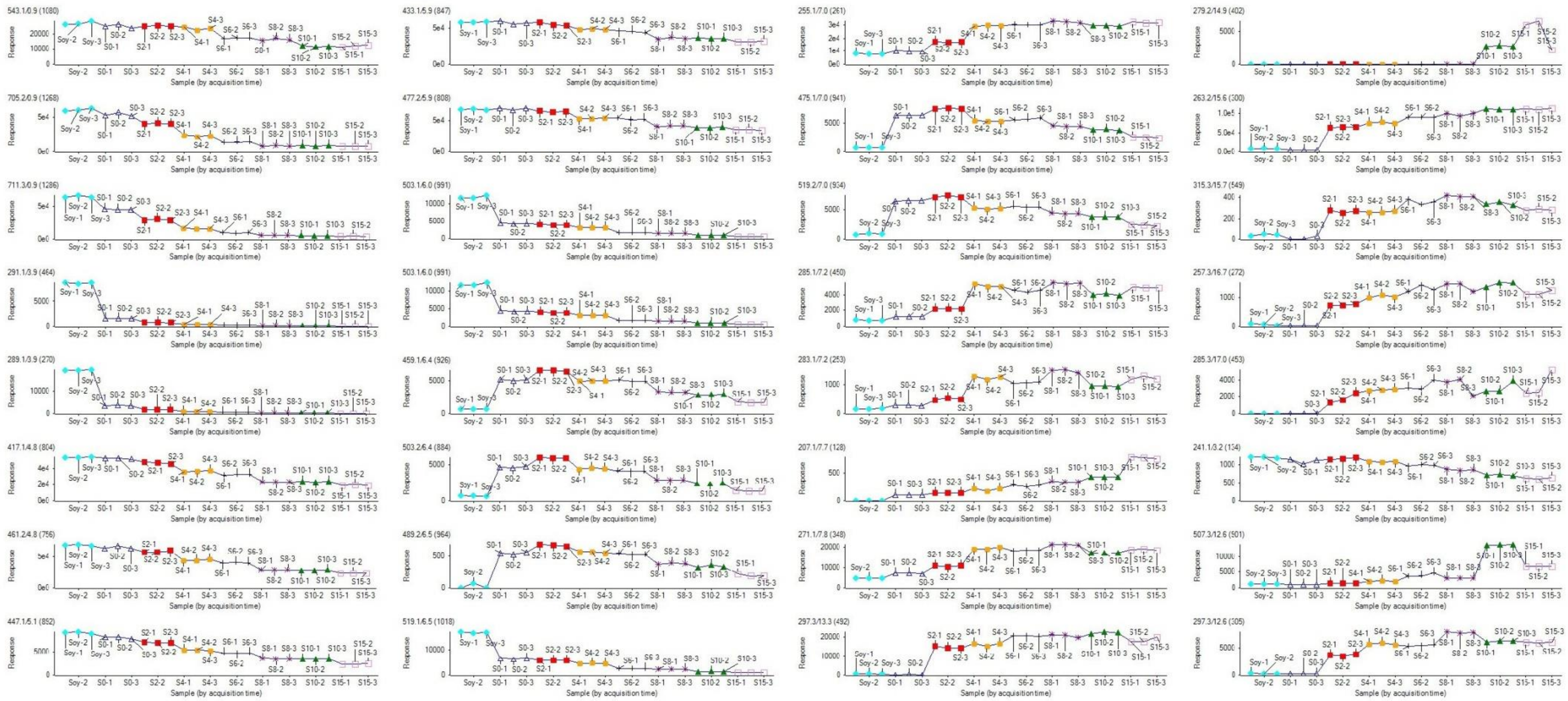

Figure 1. Selected ion intensity trend plots of assigned identities. .

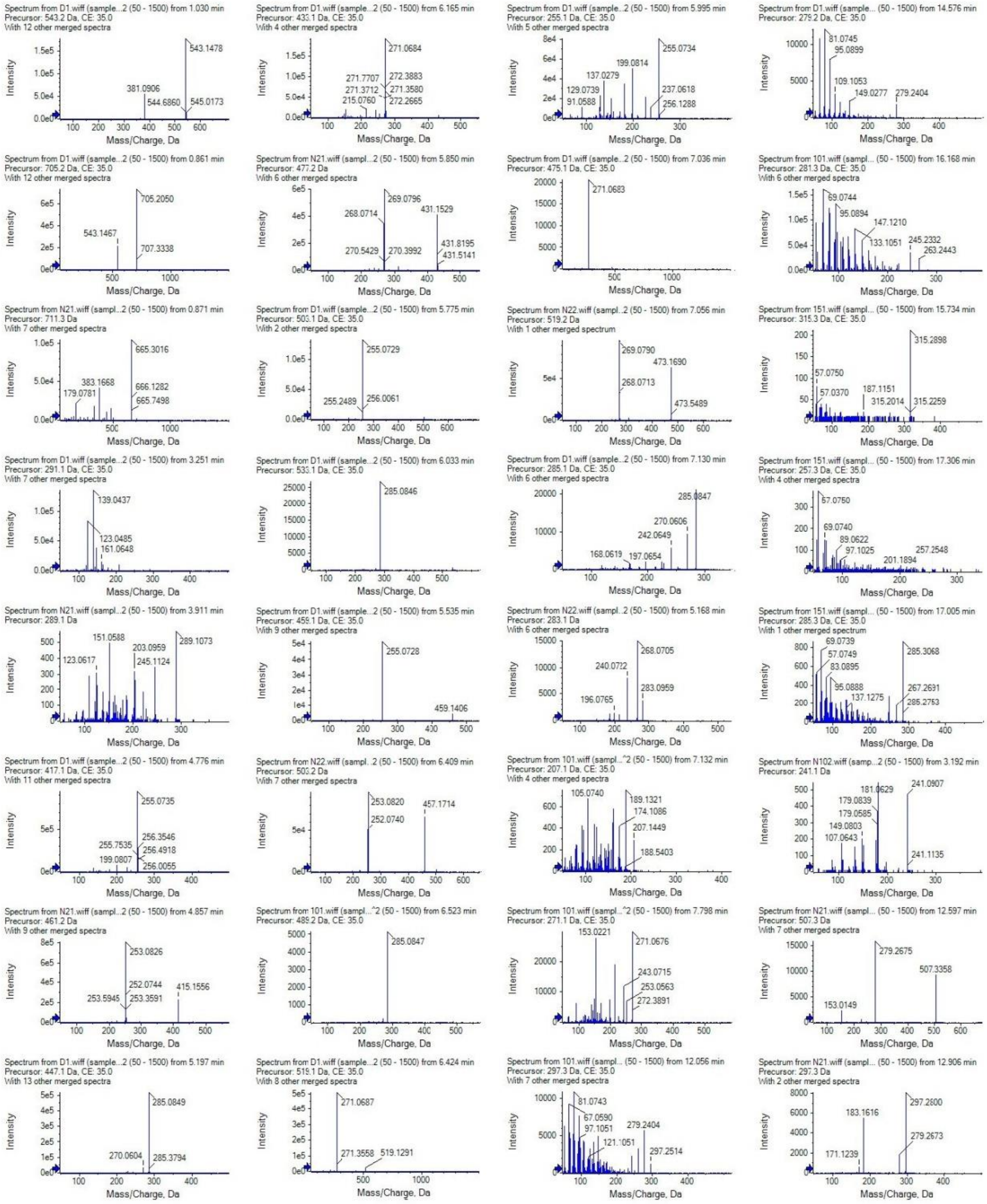

Figure S2. Mass spectrum of assigned compounds in soybean and *Semen sojae praeparatum* products.
